# Use of Innovative SPECT Techniques in the Presurgical Evaluation of Patients with Nonlesional Extratemporal Drug-Resistant Epilepsy

**DOI:** 10.1155/2021/6614356

**Published:** 2021-03-02

**Authors:** Ahmed Yassin, Khalid El-Salem, Abdel-Hameed Al-Mistarehi, Aiman Momani, Anas M. Zein Alaabdin, Palak Shah, James Michael Mountz, Anto I. Bagić

**Affiliations:** ^1^Division of Neurology, Department of Neurosciences, Faculty of Medicine, Jordan University of Science and Technology, Irbid, Jordan; ^2^Department of Public Health and Family Medicine, Faculty of Medicine, Jordan University of Science and Technology, Irbid, Jordan; ^3^Minnesota Epilepsy Group PA, St Paul, Minnesota, USA; ^4^Division of Nuclear Medicine, Department of Radiology, University of Pittsburgh Medical Center Health System, Pittsburgh, Pennsylvania, USA; ^5^University of Pittsburgh Comprehensive Epilepsy Center (UPCEC), Pittsburgh, USA; ^6^Department of Neurology, University of Pittsburgh Medical Center Health System, Pittsburgh, Pennsylvania, USA

## Abstract

Up to 30% of patients with epilepsy may not respond to antiepileptic drugs. Patients with drug-resistant epilepsy (DRE) should undergo evaluation for seizure onset zone (SOZ) localization to consider surgical treatment. Cases of drug-resistant nonlesional extratemporal lobe epilepsy (ETLE) pose the biggest challenge in localizing the SOZ and require multiple noninvasive diagnostic investigations before planning the intracranial monitoring (ICM) or direct resection. Ictal Single Photon Emission Computed Tomography (i-SPECT) is a unique functional diagnostic tool that assesses the SOZ using the localized hyperperfusion that occurs early in the seizure. Subtraction ictal SPECT coregistered to MRI (SISCOM), statistical ictal SPECT coregistered to MRI (STATISCOM), and PET interictal subtracted ictal SPECT coregistered with MRI (PISCOM) are innovative SPECT methods for the determination of the SOZ. This article comprehensively reviews SPECT and sheds light on its vital role in the presurgical evaluation of the nonlesional extratemporal DRE.

## 1. Introduction

Antiepileptic drugs are the mainstay treatment for seizures in patients with epilepsy. However, 30-40% of patients with epilepsy continue to have seizures despite optimal medical treatment [1, 2]. Complete surgical resection of the epileptogenic focus is an optimal treatment and the only potential cure in selected patients with focal Drug-Resistant Epilepsy (DRE) [3]. In such cases, accurate resection of the epileptogenic focus is imperative for successful seizure control and minimizing the risk of postsurgical neurological deficits [4, 5]. However, surgical outcomes vary considerably with respect to the etiology and the involved brain region [6–8].

For successful surgical planning, multiple tools are used to localize the seizure onset zone (SOZ), including video-electroencephalogram (V-EEG), Magnetic Resonance Imaging (MRI), Magnetoencephalography (MEG), Positron Emission Tomography (PET) scans, Single Photon Emission Computed Tomography (SPECT), and neuropsychological testing [9]. None of these tools alone has the needed sensitivity and specificity for localizing SOZ. Building a hypothesis about the SOZ based on the concordance between these tests' results is key to achieving a favorable surgical outcome [10]. DRE patients with detectable MRI lesions are 2.5-2.9 times more likely to experience a favorable surgical outcome [11]. The outcome is even more favorable when lesions are located in the temporal lobe [12]. The lack of identifiable lesion(s) on MRI, which occurs in 20-30% of patients with DRE, especially in cases of extratemporal lobe epilepsy—known as nonlesional extratemporal DRE—poses the most significant challenge [12–14]. Utilizing functional studies like SPECT can help overcome such challenge.

Here, we review the challenges associated with nonlesional extratemporal DRE and SPECT's logistical advancements and its current role in this demanding clinical scenario.

## 2. Drug-Resistant Nonlesional Extratemporal Lobe Epilepsy

Extratemporal lobe epilepsy (ETLE) may be classified as lesional when associated with a structural lesion on MR imaging, or nonlesional when there is no structural abnormality associated with EEG epileptiform abnormalities [15].

Class 1 evidence for the efficacy of epilepsy surgery came from a pivotal trial involving patients with drug-resistant temporal lobe epilepsy (TLE) who were over seven times (58% vs. 8%) more likely to be seizure-free as compared to those randomized to remain on medications only [16]. Patients with localized neocortical resections had similar success rates, but this conclusion was based on less robust studies [17]. The rates of excellent surgical outcomes for nonlesional focal epilepsy were reported in the range from 41% to 65% for the temporal lobe [18–21], 37% for mixed mesial temporal and neocortical sites [22], and 29% to 56% for ETLE [23–25]. However, Chapman et al. found that the rates of excellent surgical outcome (seizure freedom or auras only) were almost similar between TLE and ETLE (46% vs. 45%, respectively) [26].

The greatest challenge in ETLE is the localization of the SOZ [13]. Several factors could contribute to the less favorable surgical outcome when compared with TLE. These factors include the following:

1. Scalp EEG may not show changes in specific extratemporal epileptogenic focus locations, which is common in frontal lobe epilepsy [27]
2. Extratemporal onset seizures spread rapidly to other parts of the brain, obscuring the focus on EEG and possibly leading to nonlocalization or mislocalization [27]
3. Extratemporal lobe lesions (such as type I cortical dysplasia) are often not defined and discrete as temporal lobe pathology [28, 29]
4. Epileptogenic central brain regions need more conservative resection. Since central regions develop first during maturation of the brain, the developmental lesions are more abundant in these areas, and as the patient ages, these lesions tend to become more diffuse and involve eloquent brain regions. Therefore, when resection is attempted, these eloquent areas must be spared, leading to subtotal resection and so resulting in less favorable outcomes than those for TLE [28, 30]

The epileptogenic zone (EZ) is the minimum amount of cortical tissue that must be resected to produce seizure-freedom and includes the SOZ, epileptogenic lesion, and portions of the functional deficit zone [31, 32]. The EZ cannot truly be localized using imaging, and it can only be approximated post hoc pending surgical success—and confirmed using tissue analysis [31, 32]. In ETLE and neocortical TLE, the center of the epileptogenic zone varies in its location in the cortex, and the boundaries differ with every case. This makes the surgery more challenging and necessitates more investigative efforts to define the epileptogenic zone's boundaries. In contrast, mesial TLE has more consistent epileptogenic structures, and thus, more standard surgical procedures are used to remove the surgical focus [13].

Among patients who present with ETLE, those with lesions have better outcomes than those with no well-defined lesion on MRI. Despite every attempt to identify a structural lesion, some cases of ETLE will be nonlesional [33]. In those cases, the second tier of noninvasive testing may be needed and given a careful presurgical evaluation; a successful epilepsy surgery becomes possible [34].

Currently, the gold standard for presurgical evaluation of an EZ is invasive monitoring. However, this choice is limited by the invasiveness of the procedure and the need first to localize the area of interest to decrease the number of intracranial electrodes placed to avoid complications during/after surgery. The intracranial EEG also suffers from selection bias (“tunnel vision'), and therefore, its value is wholly underscored by the efficacy of the noninvasive testing in formulating correct localization hypotheses [35, 36].

The patients with refractory nonlesional ETLE pose the most challenging scenarios. Several functional imaging studies, using PET measures of metabolism or SPECT measures of regional cerebral blood flow (rCBF) changes related to interictal and ictal states at and around the EZ, have been used to assist in the localization of the epileptogenic focus [37–39].

Given the importance of SPECT as a localization tool in the process of presurgical evaluation in patients with nonlesional drug-resistant ETLE, we review the literature on its logistical advancement and practical utility in the next section.

## 3. SPECT

### 3.1. Regional Cerebral Perfusion

Regional cerebral blood flow (rCBF) and metabolism evaluation in patients with epilepsy has proven to be of significant clinical value for the localization of the epileptogenic focus. The underlying pathophysiology concerning the advantages of using regional cerebral perfusion tracers in epilepsy is based on the clinical observation that was first reported by Sir Victor Horsley more than 100 years ago [40]. Horsley described, by direct observation of the brain during surgery, an increase in cortical blood flow in the area of seizure discharge [40]. SPECT imaging is used in epilepsy mostly to provide information about regional cerebral perfusion, alteration of which is considered to depend on the regional hyper synchronization [41]. During the seizure, blood flow in the epileptogenic region can increase up to three folds [42]. This was seen as an area of hyperperfusion in an animal model [43, 44].

### 3.2. SPECT Methods and Radioligands

Since perfusion radiopharmaceuticals reach the brain within seconds after injection and the tracer molecules remain bound (with the signal loss primarily by radioactive decay of Tc-99m, 6-hour half-life), SPECT tracers can be injected at the bedside during the seizure, and imaging can be performed a short time later, allowing for patient's stabilization and transfer to the SPECT scanner. SPECT is typically performed on dual-head cameras after special care is made to optimize image acquisition parameters, image reconstruction, and image display. The subsequent scan (albeit several hours after the injection) shows the tracer uptake distribution in the brain at injection time [41, 44, 45].

**Figure 1:**
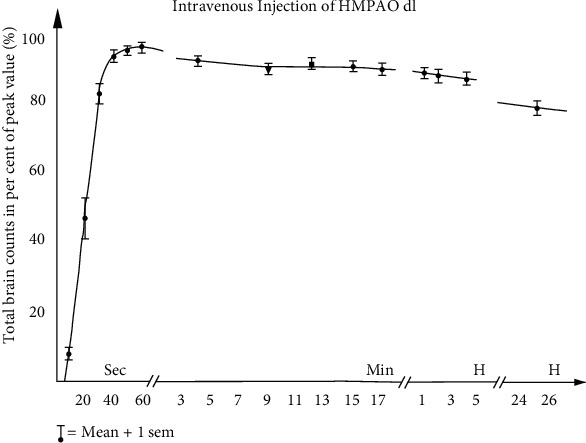
Time activity curve for Tc-99m HMPAO (corrected for radioactive decay). Note that all brain uptake has occurred by the first 60 seconds after IV injection and then very little loss of tracer molecules occurred over hours (counts decreasing mainly by radioactive 6-hour half-life decay of Tc-99m).

The ideal tracer used during the ictal SPECT study is lipophilic technetium-based (^99m^Tc). It has a rapid brain uptake across the blood-brain barrier, which is proportional to the blood flow. It also has a long retention time in the brain with minimal extracerebral uptake, and rapid clearance from blood to maximize brain on background contrast [46]. Such characteristics can help overcome the challenges mentioned above regarding localizing the SOZ in drug-resistant nonlesional ETLE [47]. Common substances used for ictal SPECT are ^99m^Tc-HMPAO (^99m^Tc-hexamethyl propylene amine oxime) and ^99m^Tc-ECD (^99m^Tc-bicisate) [48]. Both agents have similar pharmacokinetic profiles except for slightly higher extracerebral uptake of ^99m^Tc-ECD. They both have rapid brain uptake within 1 minute of the injection and are fixed in the brain by 1-2 minutes [48, 49]. (Figure 1). Both tracers are converted from lipophilic to hydrophilic polar substances after entering the cell, preventing back-diffusion from the brain cells.

Though several comparative studies failed to show a significant difference between the tracers, one study has shown that 99mTc-ECD was associated with shorter injection latencies and a higher number of accurate ictal injections compared to Tc-HMPAO, resulting in higher sensitivity and specificity in localizing the SOZ [50].

### 3.3. Ictal SPECT

Ictal SPECT, where a tracer ideally is injected immediately after the seizure onset, shows an area of hyperperfusion in the seizure focus, which is occasionally surrounded by an area of hypoperfusion [45]. This surrounding hypoperfusion area may be caused by the steal syndrome or may reflect an inhibitory zone [42].

Ictal SPECT can correctly localize the epileptogenic focus in up to 97% of cases with known unilateral temporal lobe epilepsy and up to 90% in known or suspected ETLE [51–54]. Also, resection of the SPECT focus has been found to be associated with a favorable surgical outcome [55]. The major limitation to ictal SPECT is that by the time the tracer reaches the brain, around 10-60 seconds after the injection added to the “time-lost” before starting and during the injection, the seizure activity may have dissipated or propagated. This might result in the ictal SPECT study showing the propagation pattern instead of onset, especially in seizures with a rapid spread like in ETLE [56, 57]. Therefore, early injection of the radiotracer during the seizure is imperative to capture blood flow changes in the EZ.

**Figure 2:**
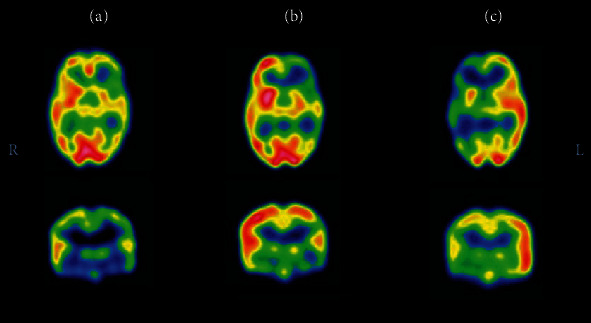
Ictal and interictal SPECT scans in a three-year-old boy with drug-resistant epilepsy. (a) Interictal SPECT showed prominent hypoperfusion over the left frontotemporal region. (b) First ictal SPECT attempt was performed in a seizure of 46-second duration, and radioisotope was injected at 40 seconds from the seizure onset. This was a late injection, and ictal SPECT showed similar findings as to the interictal SPECT. (c) Repeated ictal SPECT attempt was performed in a seizure of nine-second duration, and radioisotope was injected at two seconds from the seizure onset. This was an early injection, and ictal SPECT showed a relative increase in perfusion in the left frontotemporal region, compared with the right. This demonstrates the significance of early injection in accurately localizing the seizure onset zone, adapted from Kim et al. [38].

The postictal switch (i.e., switch from ictal hyperperfusion to postictal hypoperfusion) occurs ∼1–2 min postictally in temporal lobe seizures [48], Figure 2, but in a shorter period in extratemporal seizures. It has been estimated that extratemporal seizures should last ≥10–15 seconds after ictal SPECT injection to give precise localizing information [43]. This creates the need for an indwelling IV catheter as well as the constant and vigilant presence of medical personnel to detect the clinical or electrographic onset of the seizure to initiate injection protocol timely.

Potential reasons for failure to detect seizure focus or false localization of seizure onset include rapid seizure propagation, late injection of the radiotracer, or if the seizures are not of sufficient magnitude to produce distinguishable changes in blood flow relative to adjacent tissue using the resolution of the current scanners [58].

### 3.4. Ictal SPECT Automatic Injectors

**Figure 3:**
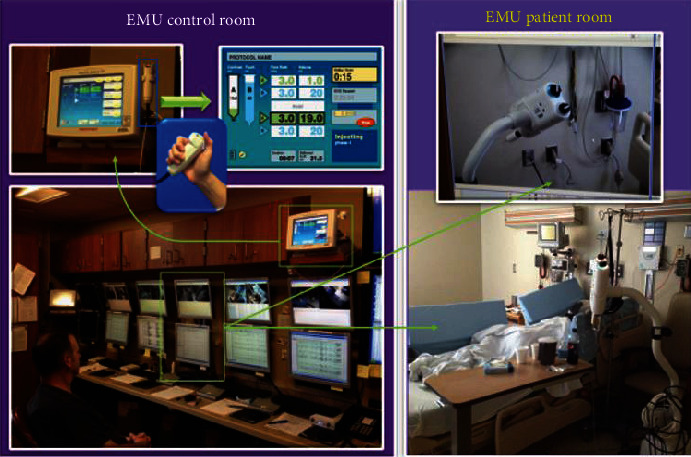
Demonstration of the use of automatic SPECT injectors at the Epilepsy Monitoring Unit (EMU) of the University of Pittsburgh Medical Center (UPMC) at Presbyterian University Hospital (PUH). Automatic injectors are armed by nuclear medicine staff. The radiotracer is injected upon a button press by the EMU staff in the EMU control room (pictures on the left side) upon observing clinical or electrographic seizure affecting the monitored patient (pictures on the right side).

One of the solutions created to improve the success in obtaining an early ictal SPECT scan was obtained by developing an automatic injector (Medrad®Spectris Solaris® EP) by Bayer's Radiology Division/Bayer HealthCare LLC/Whippany NJ, which was cleared by the FDA and became commercially available, see Figure 3. Kim et al. studied the value of using the automatic SPECT injectors in the pediatric population. This study showed a statistically significant improvement in the injection's latency time (time-lapse from seizure onset to injection initiation) [38]. Other significantly improved outcomes in the study included reducing the number of repeated studies, reducing the number of days of hospitalization, and increasing the localization rate of a single epileptogenic focus [38]. Yassin et al. did a similar study on a larger cohort of adult patients and found that automatic injectors resulted in a significant shortening of injection latency with fewer postictal injections, which led to a significant improvement in the number of successfully localizing ictal SPECTs. The latter study also showed that automatic injectors resulted in zero spills of the radiotracer and consequently represented a safer injection option for the EMU staff [59].

### 3.5. Interictal SPECT

To complete the assessment and interpretation of an ictal SPECT scan, ictal SPECT images are compared with an interictal SPECT scan. The interictal SPECT scan is performed by injecting the tracer when the patient does not have any clinical or subclinical seizures. The scan may show hypoperfusion or normal perfusion in the epileptogenic region [60]. Even when present, hypoperfusion may be mild and sometimes difficult to distinguish from the surrounding normal brain on visual examination, and thus, interictal imaging is limited in its usefulness unless combined with ictal SPECT [42, 61].

The superiority of ictal SPECT over interictal SPECT for localization of the epileptogenic focus has been demonstrated on patients with TLE, showing sensitivities between 73% and 97% for ictal SPECT and only 50% for interictal SPECT [62].

Due to the limitations mentioned earlier and low sensitivity and specificity, the role of an interictal SPECT is to assist in the evaluation of ictal SPECT, visually or quantitatively. In addition to visual comparison, the combined interictal and ictal SPECT studies may be further analyzed by using subtraction ictal SPECT coregistered to MRI (SISCOM), statistical parametric mapping (SPM), or statistical ictal SPECT coregistered to MRI (STATISCOM) [44, 63–66].

## 4. Subtraction Ictal SPECT Coregistered to MRI (SISCOM)

SISCOM uses computer-aided subtraction of interictal from ictal SPECT and then coregisters it to the MRI. In a previous study, the sensitivity of SISCOM images for localization of the EZ was 88% compared with 39% for traditional visual inspection of ictal and interictal SPECT images [48]. SISCOM can increase the focus detection rate to 92%, compared with 74% without it [67]. The sensitivity of postictal SPECT (70%–90%) has also been reported to be greater than that of interictal SPECT and can further improve with the use of SISCOM [68].

**Figure 4:**
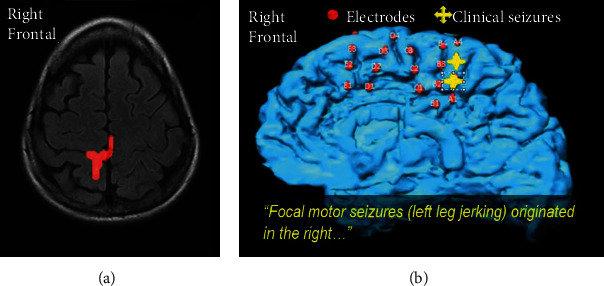
An illustrative example of a patient with distinct brief focal motor seizures involving the left leg without an EEG correlate and with a normal three tesla MRI with epilepsy protocol. SISCOM at two standard deviations (a) matched intracranial monitoring with cortical mapping (b). (a) A SISCOM (Injection latency of 6 seconds during a 14-second habitual clinical seizure without scalp EEG correlate) revealed increased uptake in the right paracentral region (leg area). (b) Cortical mapping identified the left leg motor activity in the area of seizure onset. A typical electroclinical seizure was produced by stimulation in the same region. The patient underwent responsive neurostimulation (RNS) implant leading to 100% seizure reduction.

SISCOM may be useful in localizing SOZ and guiding the extent of resection in ETLE surgery and can also predict postsurgical outcome [69].  Figure 4 demonstrates the utility of SISCOM in localizing a seizure focus in a patient with ETLE. In MRI-negative patients, SISCOM may also facilitate the detection of subtle focal cortical dysplasia (FCD) [57, 58].

For SISCOM, if no threshold for the difference is determined, there would be large hyperperfusion and hypoperfusion areas. That is why several studies have determined the area of ictal hyperperfusion by thresholding the image difference at a standard *z*-score of 1.5 or 2.0 [70–72]. Proper selection of a *z*-score (or threshold) is critical for consistent, reliable, and successful SISCOM data analysis [44, 57, 70]. SISCOM images often display multiple hyperperfusion areas, including the areas of ictal onset and seizure propagation [44]. Given the dynamic nature of SPECT, a fixed, conservative threshold may not be optimal in all patients. Some users may actually vary the *z*-score for different patients, but that depends on the reader's experience and expertise [71].

As far as limitations are concerned, SISCOM does not determine whether the ictal-interictal subtraction difference is statistically different from the expected random variation between two SPECT studies [73, 74]. SPM and STATISCOM are described in a few recent studies to overcome this drawback [74, 75].

## 5. Statistical SPECT Processing

### 5.1. STATISCOM

As mentioned above, the SISCOM does not compensate for the physiologic variance in cerebral blood flow, showing significant asymmetries in multiple areas. SPM was used in this context to determine the statistical significance of changes in perfusion in epilepsy patients in comparison to a control group without epilepsy [64–66]. SPM is a voxel-based image analysis method that involves spatial processing that combines data from various control scans and subjects. Doing that helps estimate a statistical model's parameter and make inferences about the parameter estimates with appropriate statistics. In MRI negative focal epilepsy, SPM was superior to SISCOM in localizing surgical resection site and interobserver agreement [75].

A study where ictal-interictal SPECT data were analyzed by SPM (ISAS) identified the region of seizure onset in 83% of cases with well-localized neocortical epilepsy and 71% cases with mesial temporal sclerosis [66]. Another study showed that STATISCOM was superior to SISCOM for seizure localization in TLE surgery, and localization of the STATISCOM focus to the TLE subtype was associated with a higher seizure-free outcome [74]. These studies showed improved sensitivity of SPM-SPECT for seizure focus localization. However, both sensitivity and specificity of SPM-SPECT remain unclear when used in a population of unselected patients with normal MRI focal epilepsy, like in nonlesional ETLE [74, 75].

## 6. PISCOM

Finally, a modified version of the SISCOM procedure that uses interictal PET instead of interictal SPECT for SOZ localization has recently been developed. The new processing technique known as PISCOM (PET interictal subtracted ictal SPECT coregistered with MRI) uses interictal PET and subtracts ictal SPECT and then coregisters it on MRI. There were no significant differences between this new technique and SISCOM in identifying the SOZ [76]. However, PISCOM showed a lower amount of indeterminate activity due to propagation, background, or artifacts [76]. The summary of the function and localization ability of all the functional imaging modalities mentioned above is shown in Table 1.

## 7. Conclusion

Medically refractory nonlesional ETLE is still a challenging scenario for epilepsy surgery planning with poor outcomes compared to lesional TLE. The advent of radiopharmaceutical agents to determine cerebral blood flow changes during seizures enables imaging of ictal cerebral blood flow changes. This contributes to better localization of the SOZ. This is particularly helpful in cases where MRI is negative, especially in ETLE, if there are multiple lesions with an epileptogenic potential, or if the EEG data and the imaging findings are not congruent [28]. Seizure focus localization accuracy using ictal SPECT studies can be increased with earlier injection, which can be better achieved using the automatic injectors, as well as the use of SISCOM and even more with STATISCOM. The usefulness of such studies will increase in the future as imaging methods, and analysis programs become more specialized for the purpose of seizure focus localization.

## Figures and Tables

**Table 1:** Summary of the function and localization ability of all the functional imaging modalities.

Type of SPECT study	Function	Localization rate of a seizure focus	Strengths	Limitations
Interictal SPECT	Shows hypoperfusion or normal perfusion in the epileptogenic region interictally.	50% [61, 62]	Provides a baseline interictal perfusion scan to be used for comparison with the ictal SPECT.	It cannot reliably be used alone in defining a seizure focus.
Ictal SPECT	Shows an area of hyperperfusion in the epileptogenic region, surrounded by an area of hypoperfusion, during the seizure.	Up to 97% of cases with known unilateral temporal lobe epilepsy and up to 90% with known or suspected extratemporal lobe epilepsy [51–54].	Superior to interictal SPECT in its ability to localize the seizure focus	Late injections can show areas of propagation rather than the seizure onset zone [56, 57].
SISCOM	Uses computer-aided subtraction of interictal from ictal SPECT and then co-registers it to the MRI.	88% [48] Up to 92% [67]	Increases the seizure focus detection rate and guides the extent of resection in extratemporal lobe epilepsy surgery and can also predict postsurgical outcome [67–69].	Does not determine whether the ictal-interictal subtraction difference is statistically different from the expected random variation between two SPECT studies [73, 74].
STATISCOM/SPM	Determines the statistical significance of perfusion changes in epilepsy patients by comparison to a control group without epilepsy, and so it compensates for the physiologic variance in cerebral blood flow.	71 to 83% [66] Superior to SISCOM [73–75]	Compensates for the physiologic variance in cerebral blood flow that shows significant asymmetries in multiple areas.	Both sensitivity and specificity of SPM-SPECT remain unclear when used in a population of unselected patients with normal MRI focal epilepsy, like in nonlesional ETLE [74, 75].
PISCOM	Uses interictal PET and subtracts ictal SPECT and then co-registers it on MRI.	No significant difference compared to SISCOM [76].	Showed a lower amount of indeterminate activity due to propagation, background, or artifacts [76].	The need to use two functional studies; interictal PET and ictal SPECT

## Data Availability

The data supporting this narrative review are from previously reported studies and datasets, which have been cited. They are available with the corresponding author.
